# Severe acute respiratory syndrome coronavirus 2 spike antibody level decline is more pronounced after the second vaccination, but response to the third vaccination is similar in people with type 1 and type 2 diabetes compared with healthy controls: The prospective COVAC‐DM cohort study

**DOI:** 10.1111/dom.14855

**Published:** 2022-09-15

**Authors:** Caren Sourij, Faisal Aziz, Harald Kojzar, Anna M. Obermayer, Christoph Sternad, Alexander Müller, Norbert J. Tripolt, Peter N. Pferschy, Felix Aberer, Peter Schlenke, Barbara Kleinhappl, Martin Stradner, Nazanin Sareban, Martina Moritz, Margarita Dominguez‐Villar, Nick Oliver, Ivo Steinmetz, Harald Sourij

**Affiliations:** ^1^ Division of Cardiology Medical University of Graz Graz Austria; ^2^ Interdisciplinary Metabolic Medicine Trials Unit Medical University of Graz Graz Austria; ^3^ Division of Endocrinology and Diabetology Medical University of Graz Graz Austria; ^4^ Centre for Biomarker Research in Medicine (CBmed) Graz Austria; ^5^ Department of Blood Group Serology and Transfusion Medicine Medical University of Graz Graz Austria; ^6^ Institute of Hygiene, Microbiology and Environmental Medicine Medical University of Graz Graz Austria; ^7^ Division of Rheumatology and Immunology Medical University of Graz Graz Austria; ^8^ Department of Infectious Disease Imperial College London London UK; ^9^ Department of Metabolism, Digestion and Reproduction Imperial College London UK

**Keywords:** COVID‐19, type 1 diabetes, type 2 diabetes, vaccination

## 1 CONTEXT

Up to 1 June 2022, almost 12 billion vaccine doses for coronavirus disease 2019 (COVID‐19) were administered worldwide.^1^ People with diabetes were more probable to be hospitalized for COVID‐19 and were observed to have in‐hospital mortality as high as 25%.^2^, ^3^ Hence COVID‐19 vaccination is not only recommended with priority in people with diabetes, it has also been shown to be effective in people with diabetes.^4^, ^5^

Our research group previously showed that humoral immune response 2‐3 weeks after the second COVID‐19 vaccination is similar in people with type 1 diabetes, type 2 diabetes and healthy controls,^6^ irrespective of glycaemic control. Recent data suggest that anti‐severe acute respiratory syndrome coronavirus 2 spike protein (SARS‐CoV‐2S) antibody levels decline more rapidly in people with diabetes; however, it remains unclear if there is a difference between people with type 1 diabetes and type 2 diabetes and if glycaemic control impacts the decline.^7^

The aim of this study was to analyse, first, whether the anti‐SARS‐CoV‐2S antibody rate of decline following the second COVID‐19 vaccination was impacted by diabetes status and glycaemic control and, second, if diabetes status has an impact on the response to the third COVID‐19 vaccination compared with healthy controls.

## 2 METHODS

The ‘Immune response to COVID‐19 vaccination in people with Diabetes Mellitus—COVAC‐DM’ study prospectively investigated the impact of diabetes and glycaemic control on humoral immune responses.^6^ In the main study we included adults with type 1 or type 2 diabetes, aged 18‐80 years, who were diagnosed with diabetes prior to receiving a COVID‐19 vaccine and were willing to give written informed consent. The main exclusion criteria were active malignancy (excluding intraepithelial neoplasia of the prostate gland and the gastrointestinal tract and basalioma), pregnancy, acute inflammatory disease, immunosuppressant therapy, alcohol abuse (more than 15 standard drinks a week) or any contraindication to the vaccine as well as a previous episode of COVID‐19.

Here we report the data of a substudy of participants who were willing to have the third COVID‐19 vaccination. The substudy was conducted at the study centre in Graz only.

Participants of the substudy were asked to attend on‐site visits 24 ± 2 weeks after their second vaccination and 14 to 28 days after their third vaccination. Blood samples and data on medical history, glucose‐lowering therapy, COVID‐19 infection and side effects after the third vaccination were collected. Those with a COVID‐19 infection throughout the study (symptoms suggestive of COVID‐19 and positive PCR for SARS‐CoV‐2) were excluded from the analysis.

A cohort of 67 healthy people was investigated similarly in a partner study at the Medical University of Graz (EudraCT: 2021‐001040‐10).

Antibody tests for both studies were conducted at the D&F Institute of Hygiene, Microbiology and Environmental Medicine at the Medical University of Graz. Total immunoglobulin (Ig) was determined using the Roche Elecsys anti‐SARS‐CoV‐2S electrochemiluminescence immunoassay targeting the receptor binding domain (RBD) of the viral spike protein using a Cobas e 801 analytical unit (Roche Diagnostics, Mannheim, Germany). The cut‐off for positivity is 0.8 U/ml for this assay. The study protocol of the original study was amended and approved by the ethics committee of the Medical University of Graz (33‐366 ex 20/21).

Data were analysed in Stata version 17.0 and R version 4.1.0. Categorical variables were summarized as frequency and percentage (%) and continuous variables as mean ± standard deviation (SD) or median and interquartile range (IQR) if normality assumption was violated. Normality of continuous variables was assessed using the Shapiro‐Wilk test. Quantitative variables were compared between healthy participants and those with type 1 diabetes or type 2 diabetes using either one‐way analysis of variance (ANOVA) or Kruskal–Wallis tests as appropriate. Quantitative variables were compared between type 1 diabetes and type 2 diabetes using either unpaired *t*‐test or Wilcoxon rank‐sum test as appropriate.

Changes in antibody levels over visits within each group (healthy, type 1 diabetes and type 2 diabetes) were compared using the Friedman test. Changes in antibody levels over visits between groups were assessed using multiple quantile regression with baseline antibody levels, age, sex and body mass index (BMI) used as covariates. Multiple quantile regression was used to investigate predictors of change in antibody levels in patients with diabetes. Statistical significance for each test was determined by a *P* value of less than .05.

## 3 RESULTS

Ninety‐two of the 150 participants with type 1 or type 2 diabetes of the main study who had not had COVID‐19 infection agreed to participate in this substudy.

Of the remaining 92 participants with diabetes, 44 had type 1 diabetes (19 [43%] of whom were female) with a mean age of 45.8 ± 13.3 years and a BMI of 26.1 ± 4.9 kg/m^2^, and 48 participants had type 2 diabetes (21 [44%] female) with a mean age of 58.9 ± 7.7 years and a BMI of 31.3 ± 6.2 kg/m^2^. Mean diabetes duration was 22.6 ± 14.6 years in people with type 1 diabetes and 13.8 ± 9.2 years in people with type 2 diabetes. The mean HbA1c in the group with type 1 diabetes was 7.2% ± 1.1% (55 ± 12 mmol/mol) 2 weeks after the second vaccination and 7.3% ± 1.1% (56 ± 12 mmol/mol) prior to the third vaccination, and 7.3% ± 1.1% (56 ± 12 mmol/mol) and 7.3% ± 0.9% (56 ± 10 mmol/mol) in people with type 2 diabetes, respectively.

Of the 67 healthy participants in the control group, 36 were female, the mean age was 47.2 ± 11.6 years and the mean BMI was 24.5 ± 4.2 kg/m^2^.

All participants received an mRNA‐based vaccine (the numbers of people receiving BioNTech‐Pfizer/Moderna were 39/5 in type 1 diabetes, 41/7 in type 2 diabetes and 1/66 in healthy controls, respectively).

At all time points investigated, anti‐SARS‐CoV‐2S antibody levels were above the cut‐off for positivity for the used assay in all subjects. The humoral response to the second COVID‐19 vaccination was not different between the three groups investigated 2‐3 weeks after the second vaccination ( *P* = .081).

**FIGURE 1:**
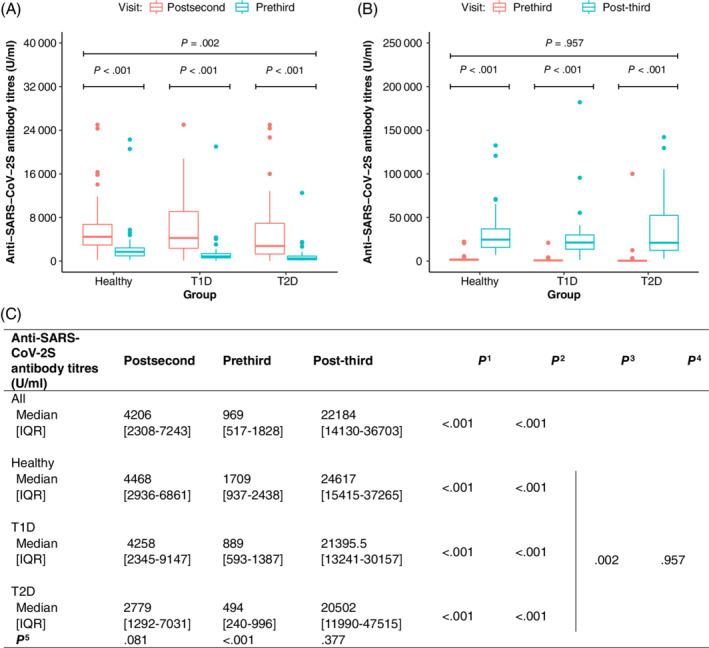
A, Median anti‐severe acute respiratory syndrome coronavirus 2 spike protein (SARS‐CoV‐2S) antibody titres post‐second vaccination and pre‐third vaccination in healthy controls, type 1 diabetes and type 2 diabetes. The top *P* value is for comparison of change in antibody levels from post‐second vaccination to pre‐third vaccination between the groups. B, Anti‐SARS‐CoV‐2S antibody titres pre‐third vaccination and post‐third vaccination in healthy controls, type 1 diabetes and type 2 diabetes. C, Anti‐SARS‐CoV‐2S antibody titres before and after vaccination. ^1^ *P* value for change in antibody levels from post‐second vaccination to pre‐third vaccination. ^2^ *P* value for change in antibody levels from pre‐third vaccination to post‐third vaccination. ^3^ *P* value for the difference in change in antibody levels from post‐second vaccination to pre‐third vaccination visit between the groups. The *P* value is adjusted for baseline antibody levels, age, sex and body mass index (BMI). ^4^ *P* value for the difference in change in antibody levels from pre‐third vaccination to post‐third vaccination between the groups. The *P* value is adjusted for baseline antibody levels, age, sex and BMI. ^5^ *P* value for the difference in antibody levels between the groups at each time point. IQR, interquartile range; T1D, type 1 diabetes; T2D, type 2 diabetes

Six months after the second vaccination, median anti‐SARS‐CoV‐2S antibody levels were 1709 (IQR 937‐2438) U/ml in the healthy control group, 889 (IQR 593‐1387) U/ml in type 1 diabetes and 494 (IQR 240‐996) U/ml in type 2 diabetes, being significantly lower in people with diabetes (*P* < .001 for both types of diabetes, adjusted for age, BMI and sex) (Figure 1A,C). When analysing the rate of decline of log‐transformed anti‐SARS‐CoV‐2S levels from post‐second vaccination to pre‐third vaccination in a regression model, again, a faster rate of decline was observed in people with type 1 and type 2 diabetes (coefficient type 1 diabetes −6.9, *P* < .001; coefficient type 2 diabetes −9.8, *P* < .001; reference group, healthy controls). However, no significant difference in the rate of decline of anti‐SARS‐CoV‐2S titres between the two types of diabetes was evident (*P* = .229).

However, the anti‐SARS‐CoV‐2S antibody response following the third vaccination was similar in people with and without diabetes (Figure **1**B).

We also investigated the impact of the vaccine used (Moderna or BioNTech/Pfizer) on the change in antibody levels following the third vaccination and did not observe a difference in the medial delta (IQR) of anti‐SARS‐CoV‐2S antibody levels, being 22 241 (13 612‐35 795) with Moderna and 18 571 (10 615‐31 196) with BioNTech/Pfizer (*P* = .135). The vaccine type was also not a significant predictor of antibody response when used in the multiple quantile regression model.Glycaemic control, as assessed by HbA1c, was neither predictive for the decline of antibody levels following the second vaccination, nor for the antibody response in the multiple quantile regression analysis (Table S1). Likewise, we did not observe a significant correlation between HbA1c and antibody response following the thirrd vaccination, both in people with type 1 and type 2 diabetes (Figures S1C and S2C).

The multiple quantile regression analysis was neither able to identify significant predictors for the decline in anti‐SARS‐CoV‐2S antibodies following the second COVID‐19 vaccination, nor for the antibody response following the third vaccination. The amount of alcohol consumption was also not predictive for the antibody response after the third vaccination (*P* = .75). Pearson correlation analyses of anti‐SARS‐CoV‐2S antibody levels post‐third vaccination and clinical and laboratory variables showed only weak correlations for age (*r* = −0.31, *P* = .043) and estimated glomerular filtration rate (*r* = 0.37, *P* = .012) in people with type 1 diabetes and for high sensitive C‐reactive protein (*r* = 0.32, *P* = .029) in people with type 2 diabetes.

## 4 CONCLUSIONS

This study shows that anti‐SARS‐CoV‐2S antibody levels fall to a lower concentration in people with diabetes 6 months after vaccination than in healthy individuals.

One reason for this observation might be attributable to the non‐enzymatic glycation and, through that, modifying of the structure and functions of immunoglobulins,^9^ potentially leading to a faster antibody turnover. The faster decline of anti‐spike antibody levels in diabetes patients may also be explained by the potential role of insulin signalling in the survival of long‐lived plasma cells.^10^ Indeed, a decreased abundance of maturation‐associated B cell phenotypes was observed in patients with type 1 diabetes.^11^

The humoral immune response to a third COVID‐19 vaccination was similar in people with type 1 and type 2 diabetes and healthy controls 2‐4 weeks after the third vaccination.

This finding is in line with our previous observation of the humoral immune response following the second COVID‐19 vaccination, which was similar in people with and without diabetes,^6^ but in contrast to another study suggesting a reduced immune response in people with diabetes,^8^ which used the GenScript SARS‐CoV‐2 surrogate virus neutralization test in contrast to the Roche Elecsys anti‐SARS CoV‐2 S assay targeting the RBD in our study, which might explain the difference in the finding.

A limitation of this analysis is that we investigated the humoral immune response only and we cannot rule out that the cellular immune response might behave differently in people with diabetes following the third vaccination. Moreover, we did adjust our analysis for age and BMI; however, cohorts of people with type 1 and type 2 diabetes are significantly different with respect to those two characteristics, hence we cannot entirely rule out an impact of those factors, despite statistical adjustment. Another limitation is that absence of diabetes was determined in the healthy control group by medical history and absence of glucose‐lowering medication only.

In summary, we believe that the data confirm our previous observation that the humoral immune response to a COVID‐19 vaccination is not hampered by diabetes. However, the decline of the antibody levels after the vaccination appears to be more pronounced in people with diabetes irrespective of glycaemic control, suggesting that shorter vaccination intervals may be necessary in both people with type 1 and type 2 diabetes, with the latter group showing the lowest antibody levels 6 months after the second vaccination.

## AUTHOR CONTRIBUTIONS

HS, F. Aziz and C. Sourij designed the study. C. Sourij and HS drafted the first version of the manuscript. HK, PNP and NJT performed the data preparation. F. Aziz performed statistics and created the figures. C. Sourij, F. Aberer, C. Sternad, AMO, HK, PNP, AM, NS and MM performed the subject recruitment and were in charge of the conduct of study visits. BK and PF performed antibody measurements. IS supervised antibody measurements. MS and PS supervised and designed the healthy control study. Data on healthy subjects were provided by MS. MDV and NO provided ciritial insight on the immunological aspects of the study. All the authors critically revised the manuscript, agreed to the submission of the latest version and contributed sufficiently to this work.

## CONFLICT OF INTEREST

The authors report no conflicts of interest related to the study.

### PEER REVIEW

The peer review history for this article is available at https://publons.com/publon/10.1111/dom.14855.

## Supporting information


Appendix S1

## Data Availability

Data is available upon reasonable request to the corresponding author.
