# Supplementary material for: Severe acute respiratory syndrome coronavirus 2 spike antibody level decline is more pronounced after the second vaccination, but response to the third vaccination is similar in people with type 1 and type 2 diabetes compared with healthy controls: The prospective COVAC‐DM cohort study
Source: Diabetes Obes Metab. 2022 Sep 15:10.1111/dom.14855. Online ahead of print. doi: 10.1111/dom.14855 (PMC9538806; doi:10.1111/dom.14855)
Supplement: Supplementary file 1 — Appendix S1 [file DOM-9999-0-s001.docx]

**COVAC-DM sub-study**

**SUPPLEMENTAL TABLE 1** Multiple quantile regression analysis of delta anti-SARS-CoV-2S antibody titres with selected predictors (n=92)

| **Predictors** | **Coefficient** | **95% CI** | **p-value** |
| --- | --- | --- | --- |
| **Change in anti-SARS-CoV-2S antibody titres from post 2^nd^ to pre 3^rd^ vaccination** | | | |
| Type of diabetes |  |  |  |
| Type 1 diabetes | Reference |  |  |
| Type 2 diabetes | -241.54 | -4206.68 – 3723.60 | 0.904 |
| Age – years | 85.07 | -94.17 – 264.32 | 0.347 |
| Gender |  |  |  |
| Female | Reference |  |  |
| Male | -302.44 | -3340.65 – 2735.77 | 0.843 |
| BMI – kg/m^2^ | -90.09 | -389.89 – 209.72 | 0.551 |
| HbA1c – mmol/mol | -32.15 | -159.57 – 95.28 | 0.617 |
| eGFR | -9.05 | -96.79 – 78.70 | 0.838 |
| Comorbidity |  |  |  |
| 0 | Reference |  |  |
| 1 | 3250.833 | -1174.72 – 7676.39 | 0.148 |
| 2+ | 4388.672 | -719.24 – 9496.58 | 0.091 |
| **Change in anti-SARS-CoV-2S antibody titres from post 2^nd^ to pre 3^rd^ vaccination** | | | |
| Type of diabetes |  |  |  |
| Type 1 diabetes | Reference |  |  |
| Type 2 diabetes | 4485.65 | -9042.395 18013.7 | 0.511 |
| Age – years | 6.59 | -604.95 – 618.12 | 0.983 |
| Gender |  |  |  |
| Female | Reference |  |  |
| Male | 5781.27 | -4584.32 16146.86 | 0.270 |
| BMI – kg/m^2^ | 682.83 | -340.04 – 1705.69 | 0.188 |
| HbA1c – mmol/mol | 258.61 | -176.12 – 693.35 | 0.240 |
| eGFR – ml/min/1.73 m^2^ | 175.80 | -123.57 – 475.17 | 0.246 |
| Comorbidity |  |  |  |
| 0 | Reference |  |  |
| 1 | -7706.95 | -22805.82 – 7391.92 | 0.313 |
| 2+ | -12868.22 | -30295.11 – 4558.67 | 0.146 |

**SUPPLEMENTAL FIGURE 1** Pearson Correlation between log of anti-SARS-CoV-2S antibody titres (post 3^rd^ vaccination) with selected variables in patients with type 1 diabetes

**
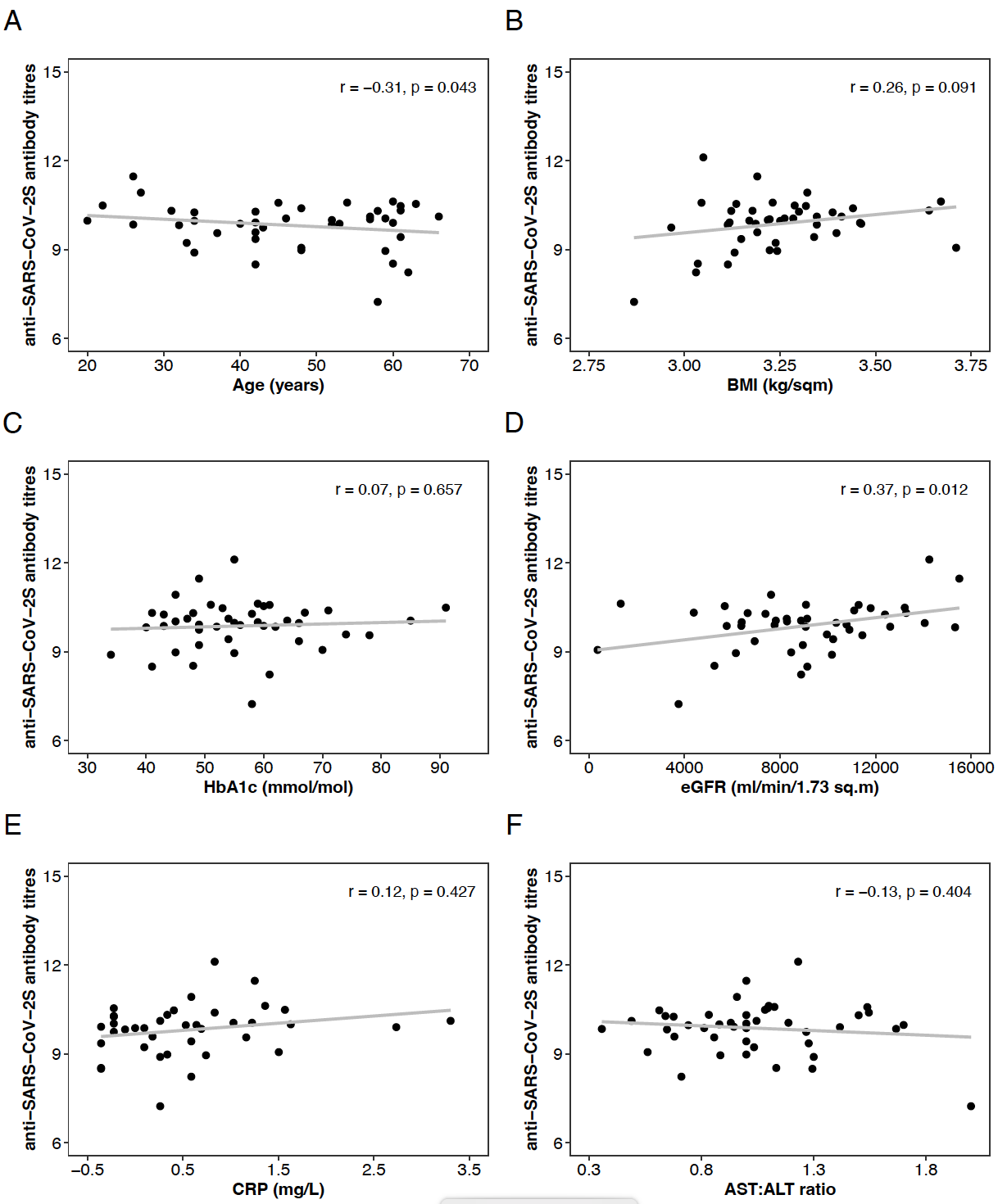
**

*BMI: log of BMI, CRP: log of CRP, eGFR: Square of eGFR

**SUPPLEMENTAL FIGURE 2** Pearson Correlation between log of anti-SARS-CoV-2S antibody titres (post 3^rd^ vaccination) with selected variables in patients with type 2 diabetes

*BMI: log of BMI, CRP: log of CRP, eGFR: Square of eGFR
